# Epidemiology and clinical characteristics of human coronaviruses OC43, 229E, NL63, and HKU1: a study of hospitalized children with acute respiratory tract infection in Guangzhou, China

**DOI:** 10.1007/s10096-017-3144-z

**Published:** 2017-12-06

**Authors:** Zhi-Qi Zeng, De-Hui Chen, Wei-Ping Tan, Shu-Yan Qiu, Duo Xu, Huan-Xi Liang, Mei-Xin Chen, Xiao Li, Zheng-Shi Lin, Wen-Kuan Liu, Rong Zhou

**Affiliations:** 1 State Key Laboratory of Respiratory Diseases, National Clinical Research Center for Respiratory Disease, The First Affiliated Hospital of Guangzhou Medical University, Guangzhou Medical University, Guangzhou, Guangdong 510182 China; 2 Department of Pediatrics, The First Affiliated Hospital of Guangzhou Medical University, Guangzhou Medical University, Guangzhou, Guangdong 510120 China; 3 Department of Pediatrics, Sun Yat-Sen Memorial Hospital, Sun Yat-Sen University, Guangzhou, Guangdong 510120 China

## Abstract

Human coronaviruses (HCoV) OC43, 229E, NL63, and HKU1 are common respiratory viruses which cause various respiratory diseases, including pneumonia. There is a paucity of evidence on the epidemiology and clinical manifestations of these four HCoV strains worldwide. We collected 11,399 throat swabs from hospitalized children with acute respiratory tract infection from July 2009 to June 2016 in Guangzhou, China. These were tested for four strains of HCoV infection using real-time polymerase chain reaction (PCR). HCoV-positive patients were then tested for 11 other respiratory pathogens. 4.3% (489/11399) of patients were positive for HCoV, of which 3.0% were positive for OC43 (346/11399), 0.6% for 229E (65/11399), 0.5% for NL63 (60/11399), and 0.3% for HKU1 (38/11399). Patients aged 7–12 months had the highest prevalence of HCoV and OC43 when compared with other age groups (*p* < 0.001). The peak seasons of infection varied depending on the HCoV strain. Patients infected with a single strain of HCoV infection were less likely to present fever (≥ 38 °C) (*p* = 0.014) and more likely to present pulmonary rales (*p* = 0.043) than those co-infected with more than one HCoV strain or other respiratory pathogens. There were also significant differences in the prevalence of certain symptoms, including coughing (*p* = 0.032), pneumonia (*p* = 0.026), and abnormal pulmonary rales (*p* = 0.002) according to the strain of HCoV detected. This retrospective study of the prevalence of four HCoV strains and clinical signs among a large population of pediatric patients in a subtropical region of China provides further insight into the epidemiology and clinical features of HCoV.

## Introduction

Respiratory viral infections in humans, which can vary from common colds to severe respiratory disease, represent a significant global health burden and a pressing public health challenge in developing countries and among socioeconomically disadvantaged children in particular. Human coronaviruses (HCoV) OC43, 229E, NL63, and HKU1 are associated with a wide range of upper respiratory tract infections (URTI) and, occasionally, lower respiratory tract infections (LRTI), including pneumonia and bronchiolitis [1–4], particularly in children [5]. Although HCoV is widespread globally [6–8], the frequency of detection of its four major strains varies significantly both by geography and over time [9–13]. Despite these features of its epidemiology, few long-term studies of the prevalence of HCoV strains and their clinical manifestations have been undertaken [14–16]. This paucity of evidence has led to an incomplete characterization of the epidemiology and clinical presentation of HCoV across different contexts.

To expand the existing evidence base and provide new insights into the epidemiology and clinical manifestations of HCoV in a subtropical region, we performed a 7-year study of four HCoV strains among hospitalized pediatric patients with acute respiratory tract infection (ARTI) in Guangzhou, China.

## Materials and methods

### Sample collection

Throat swabs were collected from pediatric patients (≤ 14 years old) hospitalized with ARTI at The First Affiliated Hospital of Guangzhou Medical University and Sun Yat-Sen Memorial Hospital from July 2009 to June 2016. Both hospitals, each with nearly 2000 beds, were located in urban areas in Guangzhou, the capital city of a province with a humid subtropical climate. A case of HCoV was defined when a patient presented at least two of the following symptoms: cough, pharyngeal discomfort, rhinobyon, rhinorrhea, sneeze, dyspnea, or diagnosed with pneumonia by chest radiography during the previous week. The respiratory samples were refrigerated at 2–8 °C in viral transport medium, transported on ice to the State Key Laboratory of Respiratory Diseases, and analyzed immediately or stored at − 80 °C before analysis.

Cases were retrospectively categorized into three groups according to their clinical symptoms: URTI, influenza-like symptoms, and LRTI. Patients presenting with cough, expectoration, rhinorrhea, rhinobyon, sneeze, pharyngeal discomfort, or trachyphonia were classified as having URTI. Patients with fever (≥ 38 °C), chills, dizziness, headache, myalgia, or debilitation were classified as having influenza-like symptoms. Patients with bronchopneumonia, pneumonia, asthma, shortness of breath, chest tightness, chest pain, or abnormal pulmonary rales were classified as having LRTI. Bronchopneumonia and pneumonia were diagnosed with chest radiography. Other clinical symptoms were identified by common medical examinations and clinical descriptions.

### Real-time PCR for HCoV detection

RNA was extracted from throat swab samples using the QIAamp Viral RNA Mini Kit (Qiagen, Shanghai, China), according to the manufacturer’s protocols. Samples were tested for four HCoV strains (HCoV-229E, HCoV-OC43, HCoV-NL63, and HCoV-HKU1) using the TaqMan real-time PCR testing kit (Guangzhou HuYanSuo Medical Technology Co., Ltd.), as previously reported [17].

### Detection of common respiratory pathogens in HCoV-positive patients

HCoV-positive samples were simultaneously tested using TaqMan real-time PCR assays (Guangzhou HuYanSuo Medical Technology Co., Ltd.) for the following 11 respiratory pathogens: influenza A virus (Flu A), influenza B virus (Flu B), respiratory syncytial virus (RSV), human bocavirus (HBoV), adenovirus (ADV), human rhinovirus (HRV), human metapneumovirus (HMPV), enterovirus (EV), *Mycoplasma pneumoniae* (MP), *Chlamydia pneumoniae* (CP), and four types of human parainfluenza virus (HPIV).

### Statistical analysis

All data were analyzed with SPSS statistical software (version 19.0; SPSS Inc., Chicago, IL), as described previously [18]. The χ^2^ test and Fisher’s exact test were used for comparisons of data. All tests were two tailed and *p* < 0.05 was considered statistically significant.

## Results

### Detection of HCoV among patients with ARTI

A total of 11,399 hospitalized pediatric patients (≤ 14 years old) with ARTI were enrolled in this study between July 2009 and June 2016. The median age of the patients was 1.75 years (interquartile range, 0.75–3.83) and the male to female ratio was 1.82:1 (7361:4038). We found that 489 out of the 11,399 patients (4.3%) tested positive for HCoV. Of these, 346 (3%) were positive for HCoV-OC43, 65 (0.6%) for HCoV-229E, 60 (0.5%) for HCoV-NL63, and 38 (0.3%) for HCoV-HKU1. The median age of HCoV-positive patients was 1.25 years (interquartile range, 0.75–3) and the male to female ratio was 1.67:1 (306:183).

### Co-infection in HCoV-positive patients

Samples from HCoV-positive patients were also tested for 11 other common respiratory pathogens. Of the 489 HCoV-positive patients, 258 (52.8%) were infected with only one HCoV strain, while 231 (47.2%) were found to be co-infected with one or more additional strains of HCoV or another respiratory pathogen (Table 1). Of these, the most frequently identified pathogens were Flu A (21.6%, 50/231) and RSV (21.6%, 50/231).

**Table 1:** Co-pathogen detection in human coronavirus-positive patients

Co-pathogen^a^	HCoV (*n* = 231)	229E (*n* = 38)	OC43 (*n* = 161)	NL63 (*n* = 33)	HKU1 (*n* = 19)
Flu A	50 (21.6)	7 (18.4)	38 (23.6)	4 (12.1)	4 (21.1)
RSV	50 (21.6)	10 (26.3)	29 (18.0)	10 (30.3)	3 (15.8)
MP	39 (16.9)	3 (7.9)	27 (16.8)	3 (9.1)	8 (42.1)
HPIV	33 (14.3)	5 (13.2)	27 (16.7)	2 (6.1)	1 (5.3)
ADV	22 (9.5)	3 (7.9)	14 (8.7)	5 (15.2)	2 (10.5)
EV	20 (8.6)	4 (10.5)	10 (6.2)	6 (18.2)	1 (5.3)
HBoV	15 (6.5)	–^c^	9 (5.6)	3 (9.1)	3 (15.8)
HMPV	15 (6.4)	3 (7.9)	14 (8.7)	1 (3.0)	–
HRV	13 (5.6)	2 (5.3)	10 (6.2)	1 (3.0)	–
Flu B	10 (4.3)	–	9 (5.6)	–	1 (5.3)
CP	4 (1.7)	–	3 (1.9)	1 (3.0)	–
HCoV^b^	18 (7.8)	15 (39.5)	16 (9.9)	4 (12.1)	2 (10.5)
229E			15 (9.3)	2 (6.1)	–
OC43		15 (39.5)		2 (6.1)	1 (5.2)
NL63		2 (5.2)	2 (1.2)		1 (5.2)
HKU1		–	1 (0.6)	1 (3)	

Data are presented as no. (%) of each group. Percentages sum to over 100% because some patients had more than one diagnosis

^a^Flu A, influenza A virus; Flu B, influenza B virus; ADV, adenovirus; HRV, human rhinovirus; HMPV, human metapneumovirus; EV, enterovirus; MP, *Mycoplasma pneumoniae*; RSV, respiratory syncytial virus; HBoV, human bocavirus; HPIV, human parainfluenza virus; CP, *Chlamydia pneumoniae*; HCoV, human coronavirus

^b^Detection of more than one strain of HCoV

^c^Not detected

### Age distribution of HCoV-positive patients

**Fig. 1:**
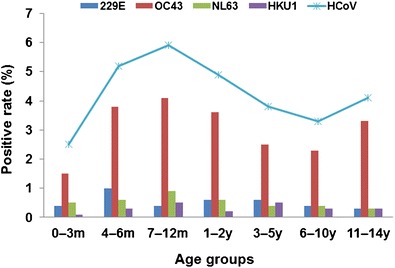
Age distribution of patients with human coronaviruses OC43, 229E, NL63, and HKU1. m: month(s); y: year(s)

In this study, patients were divided into seven age groups: 0–3 months, 4–6 months, 7–12 months, 1–2 years, 3–5 years, 6–10 years, and 11–14 years. There were statistically significant differences in the prevalence of overall HCoV and of HCoV-OC43 by age group (*p* < 0.001). Patients aged 7–12 months had the highest prevalence of both overall HCoV (5.9%, 71/1203) and HCoV-OC43 (4.1%, 49/1203) compared with the other age groups (Fig. 1). There were no significant differences in the prevalence of HCoV-229E (*p* = 0.429) or HCoV-NL63 (*p* = 0.437). Too few cases of HCoV-HKU1 were identified to assess the age distribution for this strain.

### Seasonal distribution of HCoV cases

**Fig. 2:**
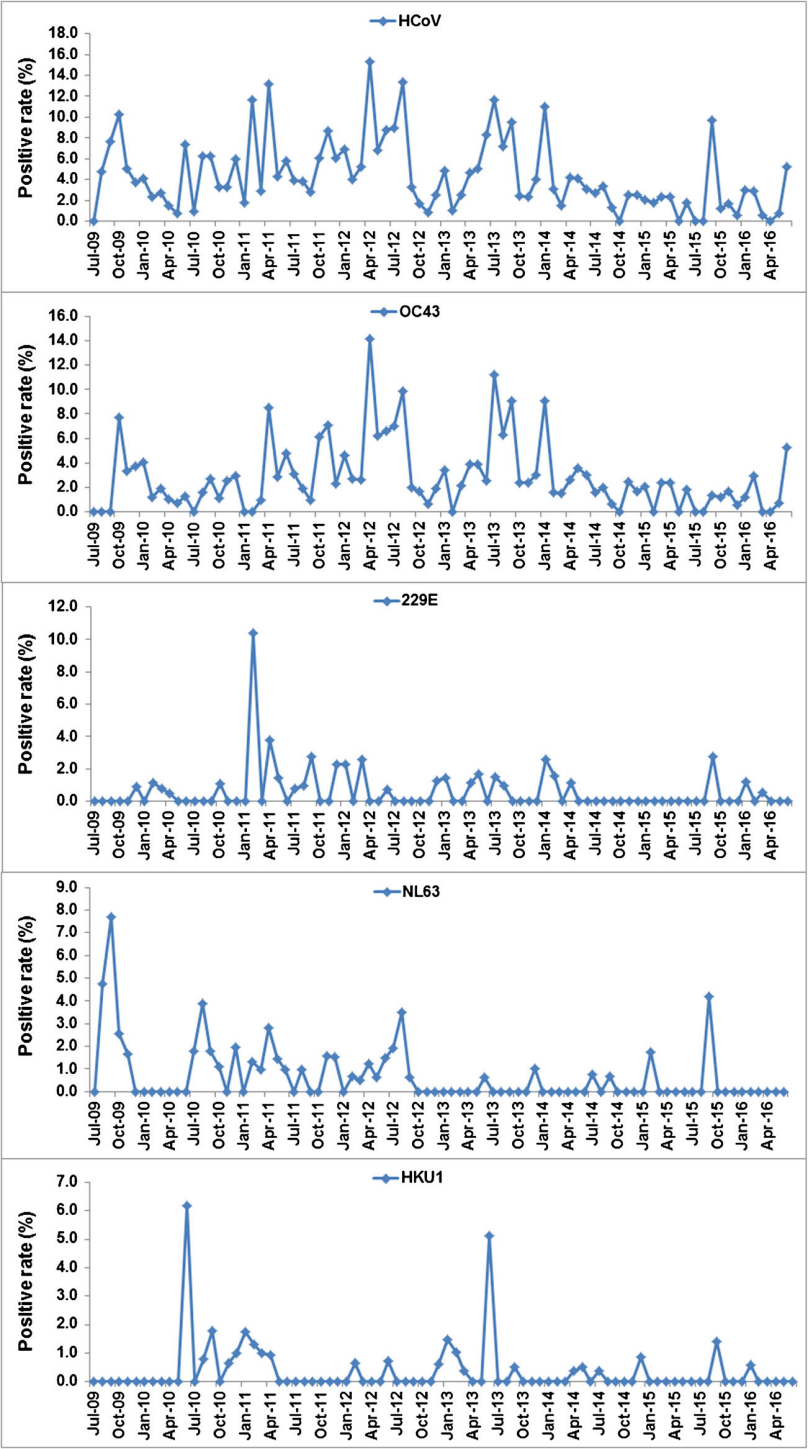
Seasonal distribution of the four human coronavirus strains in pediatric patients with acute respiratory tract infection from July 2009 to June 2016. HCoV: human coronavirus; OC43: human coronavirus OC43; 229E: human coronavirus 229E; NL63: human coronavirus NL63; HKU1: human coronavirus HKU1

There was a clear seasonal pattern in the presentation of HCoV cases over the 7-year period (Fig. 2). The overall prevalence of HCoV among attending patients tended to be highest in the spring and autumn. During the study period, the months with the highest recorded prevalences were February 2011 (11.7%, 9/77), April 2011 (13.2%, 14/106), April 2012 (15.3%, 25/163), August 2012 (13.4%, 19/142), July 2013 (11.7%, 23/196), and January 2014 (11.0%, 17/154). These seasonal trends were primarily driven by cases of HCoV-OC43. The other strains had different seasonal patterns, with smaller, more sporadic outbreaks.

### Clinical presentations of HCoV-positive patients

Table 2 shows the prevalence of clinical symptoms among HCoV-positive patients (*n* = 489) according to whether they had a single HCoV infection (*n* = 258) or were co-infected (*n* = 231), and according to the strain of HCoV detected.

**Table 2:** Clinical characteristics of human coronavirus-positive patients

Clinical presentation	Infection with HCoV			Distributions of HCoV strain				
	Single HCoV (*n* = 258)	Co-pathogen (*n* = 231)	*p*-Value^a^	229E (*n* = 65)	OC43 (*n* = 346)	NL63 (*n* = 60)	HKU1 (*n* = 38)	*p*-Value^b^
Upper respiratory tract infection								
Cough	214 (82.9)	198 (85.7)	0.401	52 (80.0)	298 (86.1)	43 (71.7)	33 (86.9)	**0.032**
Expectoration	87 (33.7)	81 (35.0)	0.755	20 (30.8)	119 (34.4)	15 (25.0)	18 (47.4)	0.137
Rhinorrhea	87 (33.7)	87 (37.7)	0.363	24 (36.9)	123 (35.6)	21 (35.0)	13 (34.2)	0.993
Rhinobyon	79 (30.6)	69 (29.9)	0.857	22 (33.9)	106 (30.6)	15 (25.0)	12 (31.6)	0.747
Sneeze	10 (3.9)	10 (4.3)	0.801	4 (6.2)	16 (4.6)	2 (3.4)	0 (0)	–
Pharyngeal discomfort^c^	17 (6.6)	15 (6.5)	0.966	8 (12.3)	22 (6.4)	4 (6.8)	3 (7.9)	0.381
Trachyphonia	9 (3.5)	0 (0)	–^e^	1 (1.5)	3 (0.9)	5 (8.3)	0 (0)	–
Influenza-like symptoms								
Fever (≥ 38 °C)	143 (55.4)	153 (66.2)	**0.014**	38 (58.5)	213 (61.6)	32 (53.3)	23 (60.5)	0.662
Chills	8 (3.1)	11 (4.8)	0.343	3 (4.6)	16 (4.6)	1 (1.7)	1 (2.6)	0.859
Dizziness	0 (0)	1 (0.5)	–	0 (0)	1 (0.3)	0 (0)	0 (0)	–
Headache	2 (0.8)	1 (0.5)	0.923	0 (0)	3 (0.9)	0 (0)	0 (0)	–
Myalgia	0 (0)	0 (0)	–	0 (0)	0 (0)	0 (0)	0 (0)	–
Debilitation	1 (0.4)	2 (0.9)	0.923	0 (0)	3 (0.9)	0 (0)	0 (0)	–
Lower respiratory tract infection								
Bronchopneumonia	47 (18.2)	52 (22.5)	0.238	12 (18.5)	79 (22.8)	8 (13.3)	6 (15.8)	0.295
Pneumonia	26 (10.1)	32 (13.9)	0.197	13 (20.0)	34 (9.8)	12 (20.0)	6 (15.8)	**0.026**
Asthma	70 (27.1)	64 (27.7)	0.887	16 (24.6)	99 (28.6)	13 (21.7)	9 (23.7)	0.632
Shortness of breath	35 (13.6)	28 (12.1)	0.634	7 (10.8)	42 (12.1)	9 (15.0)	6 (15.8)	0.776
Chest tightness	1 (0.4)	0 (0)	–	0 (0)	1 (0.3)	0 (0)	0 (0)	–
Chest pain	1 (0.4)	1 (0.4)	0.926	0 (0)	2 (0.5)	0 (0)	0 (0)	–
Abnormal pulmonary rales^d^	164 (63.6)	126 (54.5)	**0.043**	30 (46.2)	193 (55.8)	18 (30.0)	19 (50.0)	**0.002**

Data are presented as no. (%) of each group. Percentages sum to over 100% because some patients had more than one diagnosis. Significant differences in **bold**

^a^Two-tailed χ^2^ test, testing the distribution of each illness or diagnosis between patients infected with a single HCoV type and those co-infected with other type of HCoV or other respiratory pathogen

^b^Two-tailed χ^2^ test, testing the distribution of each illness or diagnosis between the four HCoV types

^c^Including pharyngeal dryness and pharyngalgia

^d^Including phlegm rale, wheeze rale, bubbling rale, moist rale, and laryngeal stridor

^e^Not performed due to small sample size

There were statistically significant differences in the prevalence of pulmonary rales and fever according to whether a patient was co-infected. While the prevalence of pulmonary rales was higher among patients with a single HCoV infection (63.6%, 164/258) than among co-infected patients (54.5%, 126/231) (*p* = 0.043), fever was more prevalent among co-infected patients (66.2%, 153/231) than those with only one HCoV strain (55.4%, 143/258) (*p* = 0.014).

There were also significant differences in the prevalence of cough (*p* = 0.032), pneumonia (*p* = 0.026), and abnormal pulmonary rales (*p* = 0.002) according to the strain of HCoV detected.

## Discussion

This retrospective study analyzed data from 11,399 hospitalized children (≤ 14 years old) presenting with ARTI in two large municipal hospitals over a 7-year period in Guangzhou, China. Given the present study’s duration and large sample size, our results represent an important addition to the evidence base on the epidemiology and clinical manifestations of HCoV. Of the 11,399 patients tested, we found that 489 (4.3%) were HCoV-positive and that the most prevalent strain of HCoV was OC43 (3.0%), followed by 229E (0.6%), NL63 (0.5%), and HKU1 (0.3%). These findings are consistent with the results of other studies around the world [11, 13, 19]. The most common co-infecting pathogens among HCoV-positive patients were Flu A and RSV (Table 1). Other recent studies have also reported that RSV, Flu A, and rhinoviruses are the most common pathogens that co-occur with HCoV, and that co-infection may influence the clinical presentation of HCoV-positive patients [4, 5, 19–21].

Consistent with studies conducted in other contexts, including America and Slovenia [16, 20], our results showed that the prevalence of HCoV was highest among patients aged 7–12 months (Fig. 1). This increased vulnerability to respiratory pathogens may be attributable to increased contact with pathogens as infants begin to explore their environment or the waning of maternal antibody levels in infants while the immune system remains underdeveloped [22–24].

HCoV is widespread globally and patterns of outbreaks vary according to locations and seasonal factors [4]. Our study found that HCoV prevalence among patients presenting with ARTI in Guangzhou over a 7-year period was highest in the spring and autumn (Fig. 2). This stands in contrast to other studies which find higher prevalence of HCoV infection in winter and spring [16, 20]. We also found different seasonal prevalence patterns for each of the four HCoV strains, with peak frequencies of 229E, NL63, and OC43 occurring mostly in the spring and autumn in Guangzhou, although OC43 had lower peaks appearing in July 2012 and 2013, however (Fig. 2). Other studies conducted in Hong Kong have shown that, while the highest frequencies of NL63 and OC43 cases occurred in autumn and winter during the period 2005–2007 [25], OC43 and HKU1 cases peaked in winter and NL63 prevalence was highest in summer and autumn during the period 2004–2005 [14]. In the United States, 229E, OC43, and HKU1 have been shown to follow different seasonal patterns, with outbreaks of 229E occurring in winter, OC43 in spring and autumn, and HKU1 in summer [20]. Although these seasonal patterns vary between countries and over time, it is apparent across all studies that the prevalence of HCoV among children is lowest in early summer.

Patients with HCoV infections presented a wide spectrum of respiratory symptoms. When we compared the clinical presentations of patients with a single HCoV infection to those with co-infections, we found that abnormal pulmonary rales occurred more frequently in the former group, while fever was more prevalent in the latter (Table 2). The results, therefore, indicate that patients infected with more than one respiratory pathogen are more likely to develop fever. Furthermore, abnormal pulmonary rales were more frequently detected among patients infected with OC43 than those infected with other strains. This suggests that HCoV-OC43 is more closely associated with LRTI. Patients with HCoV-OC43 also had the highest prevalence of broncho-pneumonia and asthma, although this was not significantly higher than among patients with other strains (Table 2). Our results are consistent with the findings of Lee and Storch [13] that HCoV-NL63 and HCoV-OC43 are associated with LRTI in children. However, Kuypers et al. [5] have found that, although HCoV-OC43 may be associated with asthma and some symptoms related to LRTI, other pathogens such as RSV may be more strongly implicated in cases of severe LRTI [26]. Recent studies have also shown that the most prevalent URTI symptoms among HCoV-positive individuals are fever, cough, sore throat, and headache [1, 19], and that LRTI including pneumonia and bronchiolitis also occasionally co-occur with HCoV [1, 5]. However, influenza-like symptoms were uncommon in our sample of HCoV-positive patients in this study.

Our study has several strengths, including its large sample size and long duration. Furthermore, given that few studies to date have simultaneously tested for all four strains of HCoV in ARTI pediatric patients, the present study addresses an important gap in the literature.

This study had some limitations, however. First, selection bias may have occurred due to the lack of healthy subjects without ARTI. Second, collecting biological samples using oropharyngeal swabs may be less reliable for detecting the presence of HCoV and other pathogens than obtaining bronchoalveolar lavage fluid. One advantage of this method, however, was that it is non-invasive and more suitable for routine analysis.

In conclusion, the four strains of HCoV investigated in the present study are common among pediatric patients with ARTI in Guangzhou, China, and are often found alongside other respiratory pathogens. HCoV infection may cause a broad spectrum of symptoms, ranging from common cold-like symptoms, to influenza-like symptoms, asthma, and even pneumonia. The present study underscores the importance of HCoV infection in the etiology of pediatric ARTI, its relevance in clinical practice, and the pressing need to improve surveillance and detection in developing country contexts.
